# Modulating satiety with neuro-music: a frequency-specific acoustic approach to appetite control

**DOI:** 10.3389/fnhum.2026.1796514

**Published:** 2026-04-27

**Authors:** Ming Chang, Takuya Ibaraki, Yoshihiro Oaku, Maki Hirate, Kenta Tanaka, Yusuke Yokota, Shin Koike, Yasushi Naruse, Yasuhiko Imamura, Shinichi Nishimoto, Manabu Abe, Tetsuya Yoshida, Yutaka Kawabata

**Affiliations:** 1 Vie, Inc., Kamakura, Kanagawa, Japan; 2 Taisho Pharmaceutical Co., Ltd., Tokyo, Japan

**Keywords:** appetite, brain rhythm, frequency-specific, monaural-auditory stimulation, neuro-music

## Abstract

**Introduction:**

Global obesity rates are rising, necessitating innovative interventions for appetite control. This study investigated whether subjective satiety—referred to as “hunger stress”—can be modulated using frequency-specific acoustic stimulation (neuro-music) designed to target neural markers associated with fullness.

**Methods:**

The research was conducted in two phases. Experiment 1 (*N* = 22) used EEG to identify neural oscillations associated with satiety and behavioral tasks to measure attentional bias toward food cues. Experiment 2 (*N* = 600) was a large-scale remote study evaluating the efficacy of neuro-music. Participants were assigned to beta/gamma, alpha, or control tracks and rated their hunger and “urge-to-eat” in two states: predining and post-snack.

**Results:**

Experiment 1 identified that satiety is characterized by significant elevations in beta (14–30 Hz) and gamma (>30 Hz) oscillations, along with a moderate increase in low-alpha (~9 Hz) power; these neural markers were correlated with reduced attentional bias toward food. In Experiment 2, although all tracks showed an initial placebo effect, only the beta/gamma neuro-music produced a prolonged suppression of hunger (*p* = 0.012) and urge-to-eat (*p* = 0.004) at 30 min post-listening. This effect was state-dependent, occurring only in the predining condition.

**Discussion:**

These findings suggest that entraining high-frequency cortical rhythms can selectively extend satiety signals during periods of metabolic need. Although the effects were transient (approximately 30 min) and did not persist after snacking, frequency-targeted neuro-music represents a promising, non-invasive tool for assisting with acute dietary regulation and appetite management.

## 1 Introduction

Rates of obesity and overweight have steadily increased over several decades, prompting significant concern regarding effective strategies to manage body weight and regulate food intake (World Health Organization (WHO), 2024; Lowe and Butryn, 2007). Traditional approaches—like cutting calories and increasing physical activity—are often challenged due to powerful physiological and psychological forces, especially the subjective feeling of hunger (Blundell et al., 2010; Freedman et al., 2001). Even people adhering to strict diets frequently struggle with persistent hunger or a persistent sense of insufficiency, which can result in lapses in self-control and overeating (Rolls, 2007). As a result, there is a pressing need for novel interventions that address not only the physical aspects of appetite control (Hall et al., 2011; Montani et al., 2015) but also the subjective, cognitive, and emotional components of satiety (Blundell et al., 2010; Lowe and Butryn, 2007; Higgs et al., 2017). Indeed, feelings of “emptiness” or the desire to eat can persist after consuming an adequate amount of food—often driven by hedonic rather than homeostatic mechanisms—posing significant hurdles for anyone trying to manage their intake (Lowe and Butryn, 2007; Monteleone et al., 2012).

Considering this challenge, nonpharmacological methods to influence the subjective sense of fullness and promote healthier eating are being developed. Recent advances in neuroscience and psychophysiology have deepened our understanding of how external sensory inputs—particularly auditory cues—can shape cognitive and emotional states by modulating the underlying neural processes (Koelsch, 2014; Miranda et al., 2011). For example, neural entrainment, where the brain’s electrical activity aligns with periodic external stimuli, can cause shifts in attention, mood, and physiological responses (Barbaresi et al., 2024; Lakatos et al., 2008). For example, Lane et al. (1998) reported that exposure to specific binaural beat frequencies produces measurable changes in electroencephalographic (EEG) patterns, which in turn affected mood and alertness. Similarly, Nozaradan (2014) showed that the brain’s oscillatory activity can get attuned to certain rhythmic structures—as demonstrated by EEG phase-locking—thereby influencing perception and motor coordination. Moreover, Thaut et al. (1996) demonstrated that rhythmic auditory stimulation can improve gait patterns in people with Parkinson’s disease, illustrating how targeted periodic stimuli can synchronize and modify both neural and physiological functions.

Beyond these examples of entrainment, extensive research has investigated the broader impacts of music. Extensive research confirms that music significantly modulates autonomic and emotional states, as reflected in changes in heart rate, respiration, and galvanic skin response (Bernardi et al., 2006; Juslin and Västfjäll, 2008; Kulinski et al., 2022; Mir et al., 2021; Stewart et al., 2019). Furthermore, neurophysiological studies demonstrate that specific auditory interventions can activate reward-related neural circuits and entrain brainwave activity, thereby shaping subjective experiences ranging from relaxation to alertness (Blood and Zatorre, 2001; Fachner et al., 2013; Chang et al., 2024).

Currently, growing evidence suggest “neuro-music” and other specialized sound-based therapies can serve as tools to modulate particular EEG frequency bands, thereby influencing emotional and cognitive states (Koelsch, 2014; Fachner et al., 2013; Chang et al., 2024). On the other hand, neuroimaging and psychophysiological studies have identified unique neural signatures tied to hunger and satiety, revealing a direct connection between measurable brain activity and subjective feelings of fullness (Haase et al., 2009; Goldstone et al., 2009). To understand how sound frequencies could regulate these states, it is necessary to consider the interaction between cortical activity and homeostatic centers. Appetite is governed by a complex network of peripheral, gastrointestinal, and central nervous system neurotransmitters and neurohormones that act primarily on the hypothalamus. Importantly, this hypothalamic regulation is intricately linked with top-down cortical activity, where sensory processing—such as audition—and cognitive control occur. Prior studies demonstrate that acoustic environments can modulate these networks; for example, ambient sound and cognitive regulation interact to alter the neurophysiology of food cravings (Peng-Li et al., 2022). By combining EEG frequency modulation with existing appetite regulation models, researchers are investigating nonpharmacological methods to support weight management and reduce stress from dieting. Though at an early stage, this interdisciplinary blend of neuroscience, psychophysiology, and experimental psychology underscores the promise of novel interventions to reshape how we perceive fullness and foster healthier eating behaviors.

In this study, we define satiety as decreased “hunger stress,” as minimal urge to eat despite the absence of full stomach distension. Based on the hypothesis that specific external auditory stimulation may help induce or reinforce such a state, our primary aim was to design and evaluate neuro-music to enhance the brain oscillations associated with satiety.

To explore this possibility, in Experiment 1, we measured and compared electroencephalographic (EEG) activity during hunger and postprandial conditions to identify frequency bands reliably associated with the satiated state. We hypothesized that one or more frequency components would be more prominent in the satiated state. These frequencies were then embedded into a custom neuro-music composition. Subsequently, in Experiment 2, we assessed whether this neuro-music could modulate subjective feelings of hunger.

## 2 Experiment 1

### 2.1 Materials and methods

#### 2.1.1 Participants

A total of 22 healthy adults (half men, age 22–58, mean = 39) were recruited for Experiment 1. Inclusion criteria required participants to be healthy individuals aged 20–59 years with normal or corrected-to-normal vision and hearing. Exclusion criteria included: (1) metabolic diseases (e.g., diabetes, hyperlipidemia) or severe obesity (BMI ≥ 30); (2) a history of eating disorders; (3) history of neurological or psychiatric conditions, including epilepsy, brain diseases, and migraines; (4) current use of psychotropic or other medications; (5) inner ear diseases (e.g., Ménière’s disease) or the use of hearing aids; and (6) allergies to metal/alcohol or an inability to maintain a seated position for extended periods. Each provided written informed consent in accordance with the principles of the Declaration of Helsinki. The experiment was approved by the Shiba Palace Clinic Ethics Review Committee (Approval number: 156951_rn-38708). To ensure a standardized fasting condition, participants were instructed to refrain from caloric intake for ≥ 10 h before each experimental session; however, they were allowed to consume noncaloric beverages (e.g., water or unsweetened tea).

#### 2.1.2 Procedure

This experiment was conducted over five sessions (Day 1–5), typically on consecutive or near-consecutive days. Before Session 1, each participant selected 10 images of their favorite foods—items perceived as most likely to induce hunger. Each session consisted of three main phases: Predining, Dining, and Postdining. At Predining and Postdining phases, participants completed the same set of activities, namely Resting-State EEG and the Food Probe Task, as well as visual analog scale (VAS) ratings after each activity.

(i) Resting-state EEG

Participants were seated comfortably with their eyes closed for 3 min while EEG data were recorded. This resting-state measurement served as baseline for subsequent comparisons. Immediately after, participants completed a VAS to assess current hunger and fullness.

(ii) Food probe task

**Figure 1:**
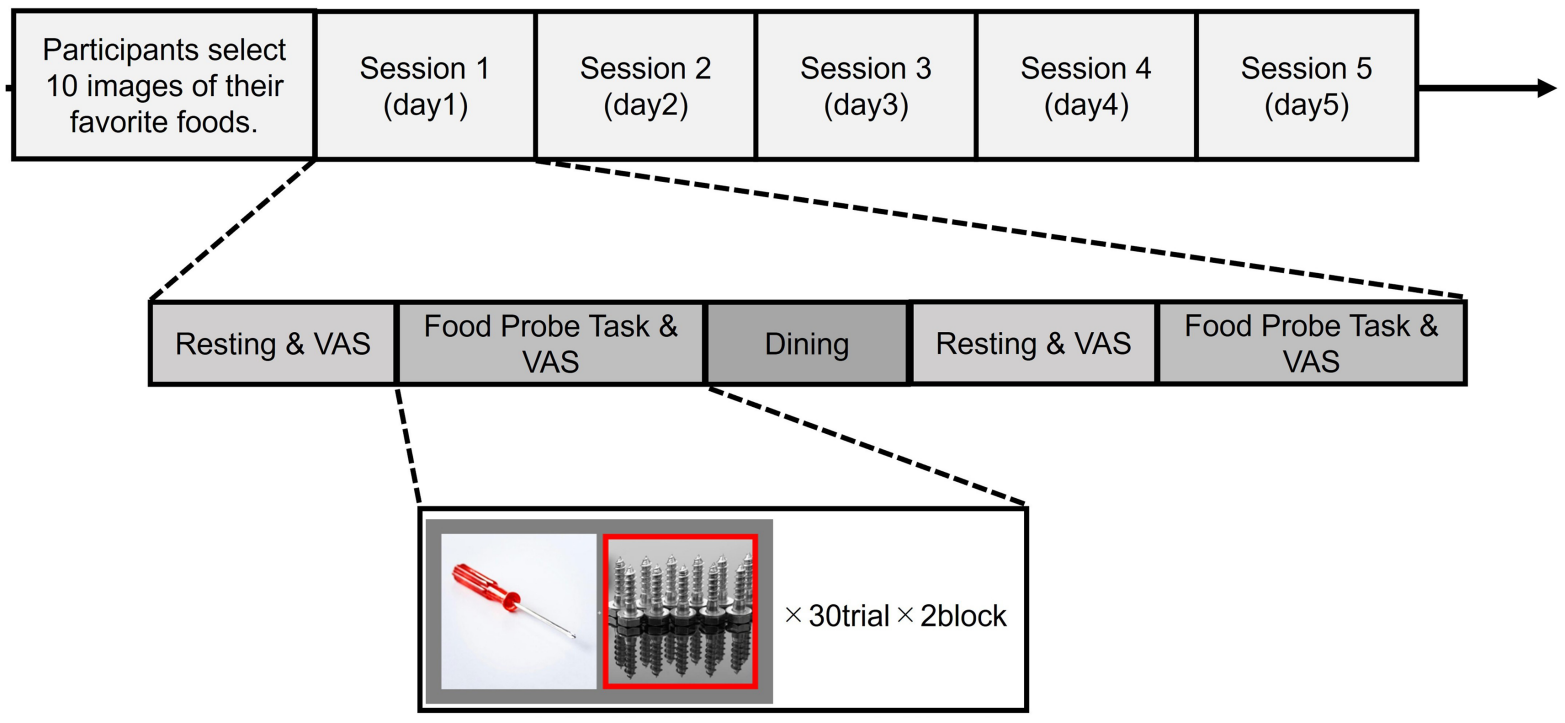
Flowchart of experimental design for Experiment 1.

Before Session 1, each participant selected 10 images of their personal favorite foods, specifically chosen to induce craving. Neutral “tool” images (e.g., hammers, screwdrivers) were included as control images. In each trial of the Food Probe Task, two images were displayed side by side on a monitor for 4 s (Figure 1). One image was always on the left, and the other on the right. After the 4-s interval, one of the two was outlined in red. Participants were instructed to respond as quickly as possible using the keyboard—pressing the “C” key if the outlined image appeared on the left, or the “M” key if on the right. Three possible image pairings were presented—Food/Neutral, Neutral/Food, or Neutral/Neutral—each pairing appearing randomly at an equal probability. The total number of trials per session was divided into two blocks (e.g., ~30 trials each), and EEG signals recorded continuously throughout the task. This design allowed for direct comparison between responses to food-related and neutral stimuli, as well as capturing any shifts in attention or engagement when food images were present. This task was implemented using software developed with the psychophysics toolbox PsychoPy and MATLAB, which allowed precise control over stimulus presentation and response recording. Immediately after completing the Food Probe Task, participants rated their hunger and fullness using a brief VAS.

(iii) VAS ratings

The VAS was used to assess subjective hunger and satiety levels. Participants rated the following three items:

- “I am extremely hungry right now”
- “I feel very full right now”
- “I want to eat something immediately”

Each item was presented as a 100-mm horizontal line, anchored with “Not at all” on the left (0 mm) and “Extremely” on the right (100 mm). Participants responded by moving the mouse cursor along the scale and clicking the left mouse button at the point that best represented their current feeling.

Then, participants took a 30-min lunch break to consume a meal they brought with. This break aimed to allow participants to shift from a hungry to a satiated state, enabling within-subject comparisons before and after eating.

After the lunch break, the participants entered the postdining phase, repeating the same activities as predining. Each session lasted approximately 1 h in total.

#### 2.1.3 EEG recordings

EEG signals were recorded using a wireless biosignal amplifier (Polymate Mini AP108, Miyuki Giken Co., Ltd., Tokyo, Japan) with solid gel electrodes (METS INC., Chiba, Japan). Following the international 10–20 system, we placed electrodes at Cz, Pz, C3, C4, F3, and F4 at a sampling rate of 500 Hz. Ground and reference electrodes were positioned on the left and right mastoids, respectively. To capture eye blinks and vertical eye movements, an additional electrode for electro-ocular (EOG) activity was placed at the upper-outer corner of the left eye.

In each experimental day, EEG signals were analyzed separately for the predining and postdining conditions. The analysis aimed to quantify relative spectral power to enable comparisons across participants, days, and conditions by minimizing individual variability and session-specific fluctuations.

The preprocessing and analysis pipeline consisted of the following steps: (1) Epoching: EEG data were segmented into 4-s windows to ensure consistent temporal resolution for spectral analysis. (2) Noise rejection: Epochs containing artifacts were excluded based on an amplitude threshold criterion. Specifically, any EEG segment with signal amplitude > ±50 μV was considered contaminated by noise (e.g., muscle activity, movement artifacts) and excluded from further analysis. (3) Spectral power calculation: A Fast Fourier Transform was applied to each artifact-free epoch to compute the absolute power spectrum. (4) Relative power (RP) computation: To normalize the data, the RP was calculated by dividing the power at each 1 Hz frequency bin (1–40 Hz) by the total power within the 1–40 Hz range for that epoch. This yielded a normalized power distribution, facilitating cross-condition and cross-participant comparisons.

To evaluate differences in RP between the predining and postdining conditions, Cohen’s *d* was calculated as an effect size measure. This enabled quantification of the magnitude of change across conditions independent of sample size and provided a standardized metric for interpreting neural changes associated with satiety.

#### 2.1.4 Statistical analysis

For the analysis of resting-state EEG data, spectral analysis was conducted on resting-state EEG data for each electrode channel. For every participant, RP was calculated at 1 Hz intervals within the 1–40 Hz frequency range and averaged across the five experimental days for each condition: Predining and Postdining. Further, Cohen’s *d* effect sizes were computed at each frequency bin to estimate the within-subject magnitude of change between conditions and subsequently averaged across days.

At group level, condition-related differences were evaluated by calculating mean differences in RP between the predining and postdining states. To assess the statistical significance of these within-subject changes across the frequency spectrum, paired-samples *t*-tests were conducted on the RP values at each 1 Hz frequency bin for each channel. Additionally, Cohen’s *d* values were calculated to estimate the standardized effect size of the magnitude of change.

For the EEG data collected during the food probe task, the spectral power was analyzed across four conventional frequency bands: theta (4–7 Hz), alpha (8–13 Hz), beta (14–29 Hz), and gamma (30–40 Hz). The RP in each band was normalized to the total spectral power within the 1–40 Hz range.

To examine the effects of internal state and stimulus category on oscillatory brain activity, a three-way repeated-measures ANOVA was performed. Within-subject factors included frequency band (theta, alpha, beta, gamma), hunger condition (hungry vs. full), and stimulus type (food vs. neutral). This analysis allowed us to assess both the main effects of each factor and potential interactions, shedding light on how physiological and perceptual influences jointly shape neural responses during food-related attentional processing. A significance threshold of *p* < 0.05 was applied, with corrections for multiple comparisons where necessary.

In addition, to compare reaction times (RTs) to food images between hungry and full conditions, a *t*-test was conducted, and Cohen’s *d* calculated to estimate the effect size. In addition, Cohen’s *d* effect sizes were calculated for VAS scores between hungry and full conditions. To further explore the relationship between subjective hunger and behavioral performance, a regression analysis was conducted using RTs and VAS scores.

### 2.2 Results

#### 2.2.1 Changes in resting-state EEG power between predining and postdining conditions

**Figure 2:**
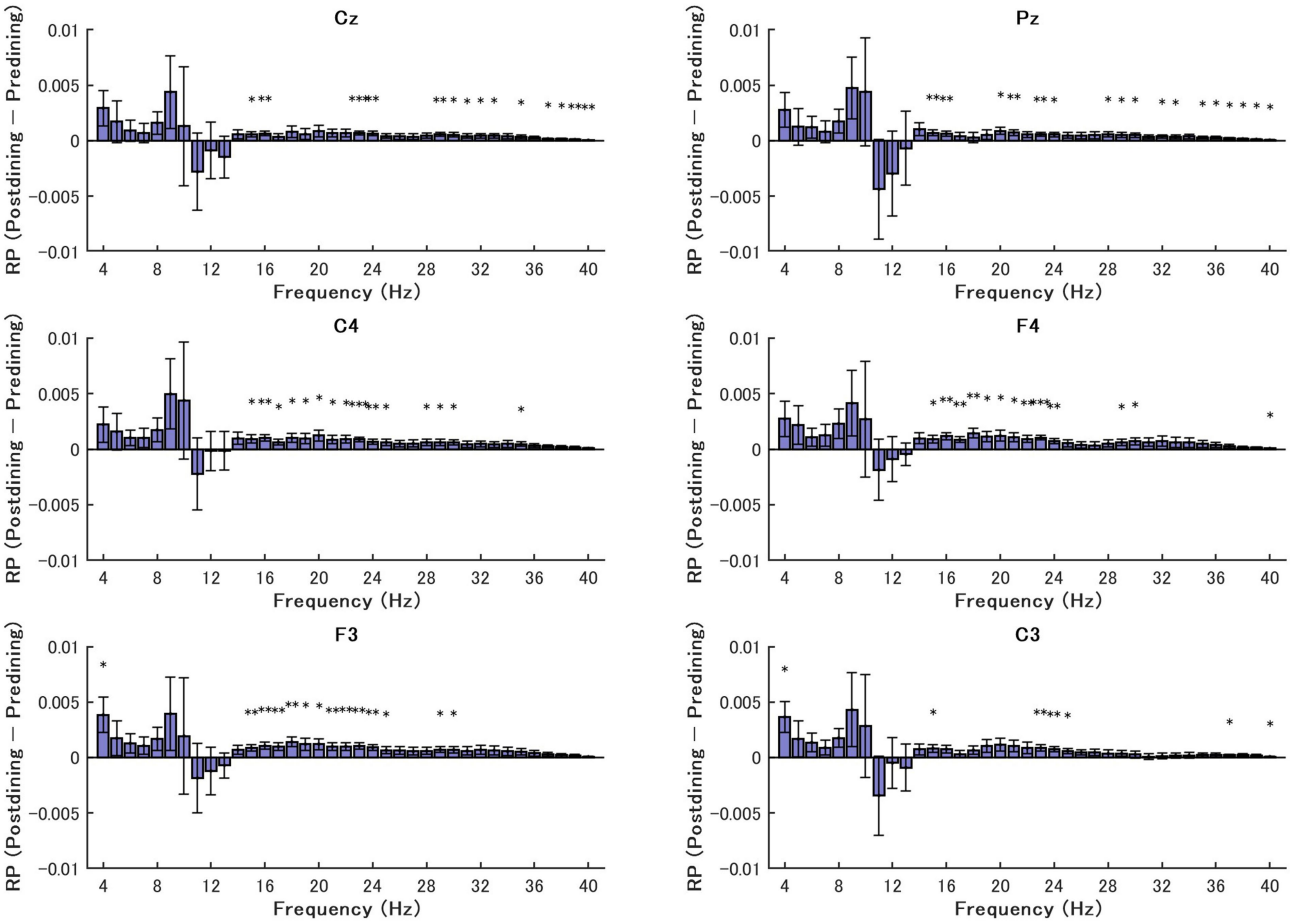
Differences in relative EEG power (postdining–predining) across frequencies (4–40 Hz) on six electrode sites (Cz, Pz, C3, C4, F3, F4) at resting state. Each panel shows the mean difference in relative power across participants, with positive values indicating greater power in the postdining condition compared to the predining condition. Error bars indicate the standard errors of the mean differences.

Figure 2 illustrates the difference in RP between postdining and predining conditions across six electrode sites.

Across all channels, a consistent pattern was observed in the theta (4–7 Hz), low alpha (8–10 Hz), beta (14–29 Hz), and gamma (30–40 Hz) bands. In these frequency ranges, RP tended to be higher in the postdining condition compared to the predining condition. Conversely, in the high-alpha range (10–13 Hz), RP was notably greater in the predining condition, appearing as a negative deflection in the difference plot. This suggests that distinct sub-bands of alpha may respond oppositely to metabolic states. Notably, within the lower frequency range, a distinct visual peak in the mean difference occurred around 9 Hz. Although this specific difference did not reach statistical significance in the subsequent paired-samples *t*-test (as detailed in Section 2.2.2), this visual trend suggests that this low-alpha frequency may be sensitive to physiological hunger.

Conversely, the topographical distribution of these effects was relatively uniform, with no strong differences across EEG channels.

#### 2.2.2 Effect sizes of frequency-specific differences between predining and postdining conditions

**Figure 3:**
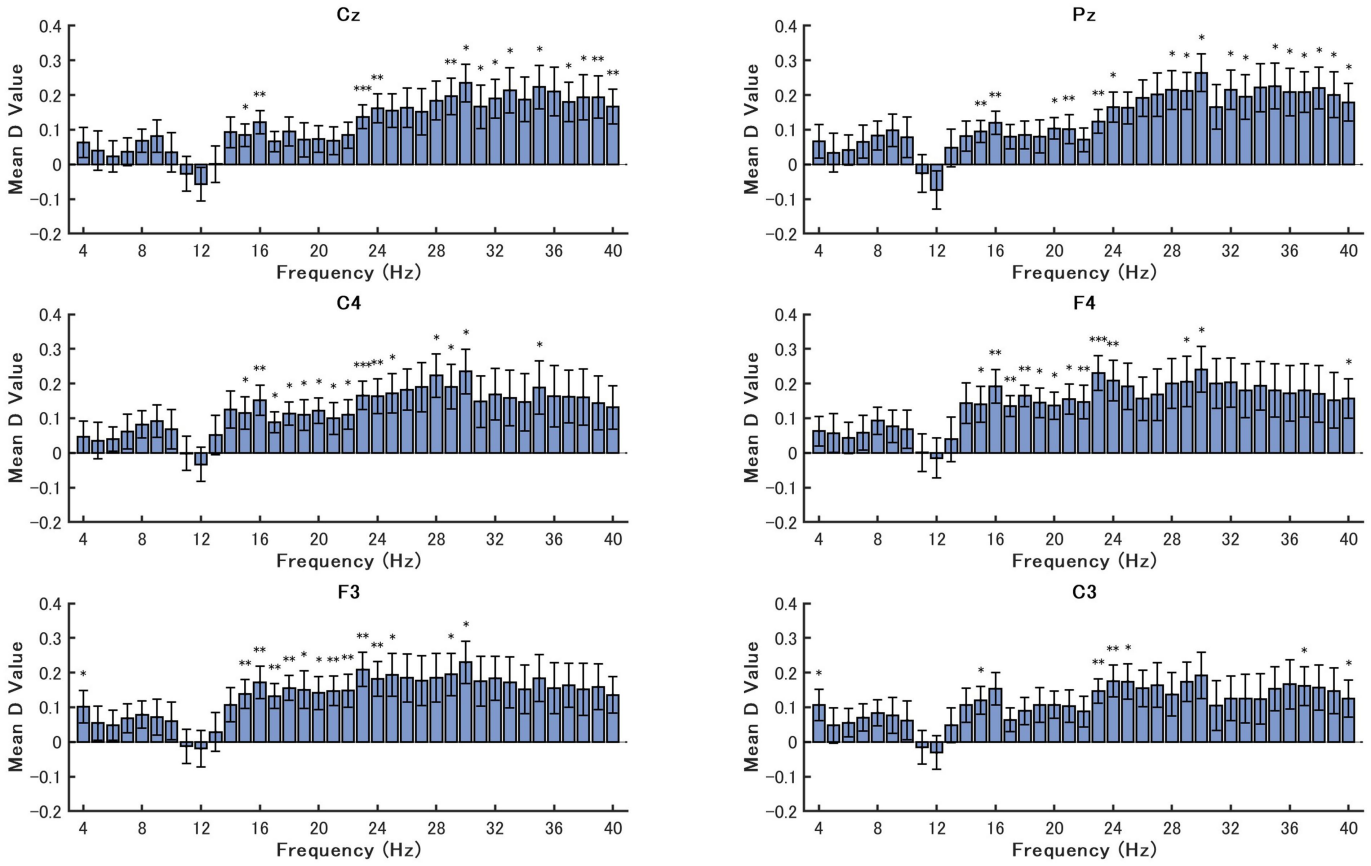
Mean Cohen’s *d* values for the differences in relative power (postdining–predining) across frequencies (4–40 Hz) at six electrode channels (Cz, Pz, C3, C4, F3, F4). Bars indicate mean effect sizes across participants and error bars indicate standard errors. Asterisks indicate significant differences between the postdining and predining conditions based on paired-samples *t*-tests (^∗^*p* < 0.05, ^∗∗^*p* < 0.01, ^∗∗∗^*p* < 0.001).

The average Cohen’s *d* values across participants for each 1 Hz frequency bin (4–40 Hz) in the resting-state EEG are shown in Figure 3. The results indicate six representative electrode sites revealing significant increases in RP in the postdining condition.

Across all channels, there was a clear pattern of positive effect sizes, most notably in the beta and gamma frequency ranges. The most prominent, widespread significant effects emerged between 24 and 36 Hz, with many channels—particularly Cz, Pz, F3, and F4—showing *p*-values < 0.05 or < 0.01 across multiple consecutive frequency bins. While the absolute values of Cohen’s *d* in these target ranges peaked near 0.25, they generally represent a small effect size. This is common in resting-state EEG due to high baseline physiological variance. Nevertheless, the high statistical significance derived from the paired-samples *t*-tests indicates that the direction of the spectral shift (i.e., increased beta/gamma) was highly consistent across individuals, confirming these changes as reliable neural markers of the postdining state. In addition, while moderately positive *d* values were visually observed in the low alpha range (8–9 Hz), these differences did not reach statistical significance under the paired-samples *t*-test. Importantly, all channels displayed a broadly similar pattern, suggesting that the topographical distribution of these effects did not exhibit strong regional specificity.

#### 2.2.3 Modulation of EEG band power during the food probe task

**Figure 4:**
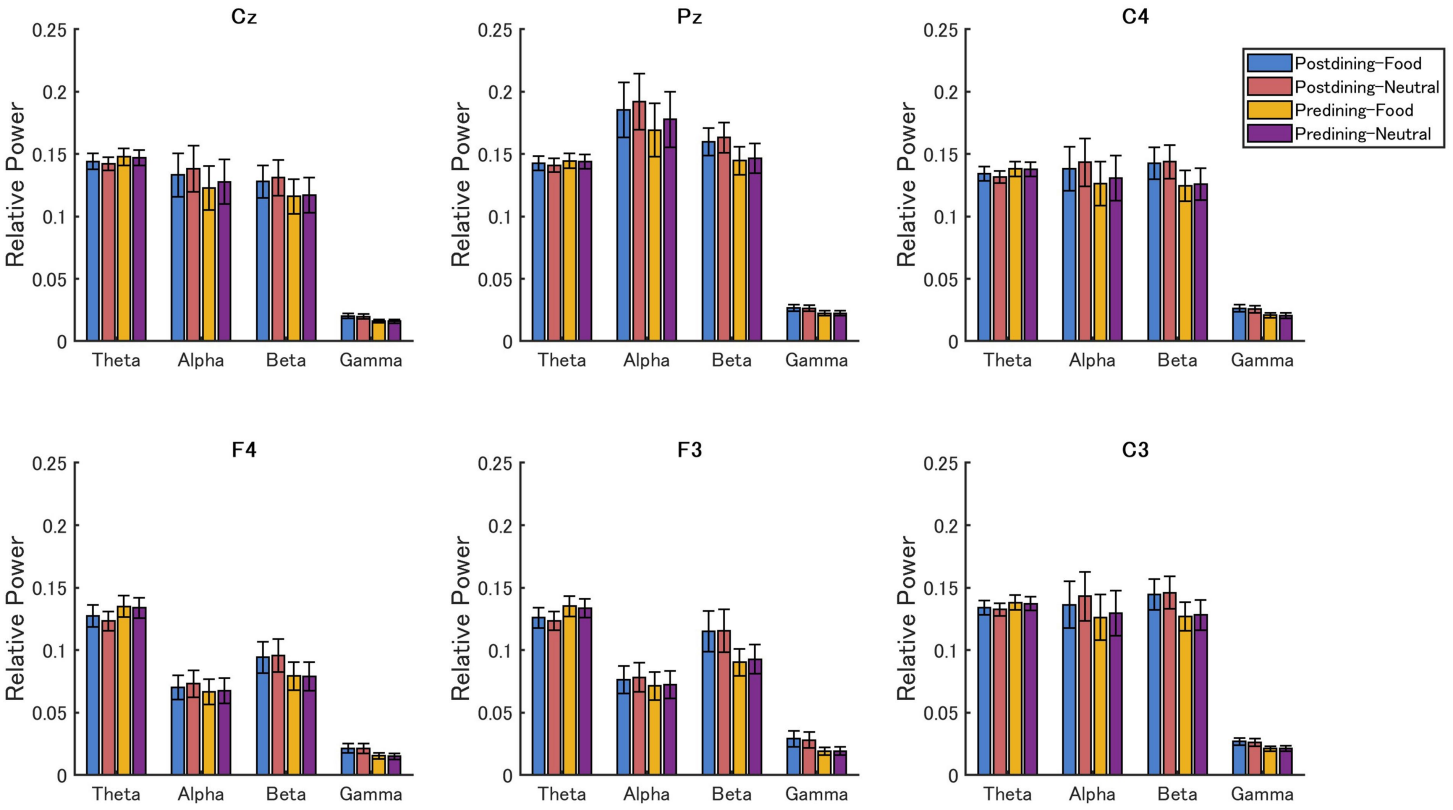
Mean relative power across frequency bands (theta, alpha, beta, gamma) under different conditions (predining vs. postdining) and stimulus types (food vs. neutral) at six electrode sites (Cz, Pz, C3, C4, F3, F4).

To assess the effects of various factors on oscillatory brain activity during the food probe task, a three-way repeated-measures ANOVA was conducted with band (theta, alpha, beta, gamma), condition (predining vs. postdining), and stimulus type (Food vs. Neutral) as within-subject factors. The results are summarized in Figure 4.

A significant interaction between frequency band and condition was observed across all channels (C3: *F*(3, 63) = 9.22, *p* < 0.001; C4: *F*(3, 63) = 10.22, *p* < 0.001; Cz: *F*(3, 63) = 9.25, *p* < 0.001; Pz: *F*(3, 63) = 8.10, *p* < 0.001; F3: *F*(3, 63) = 8.09, *p* < 0.001; F4: *F*(3, 63) = 11.78, *p* < 0.001). Post-hoc analyses revealed that the RP in the alpha, beta, and gamma bands was significantly higher in the postdining condition than in the predining condition (*p* < 0.01).

Further, post-hoc analyses showed a significantly higher RP in alpha, beta, and gamma bands in the postdining condition than in the predining condition across all channels. Specifically, alpha power increased at Cz, Pz, C3, and C4 (*p* < 0.01) and at F3 and F4 (*p* < 0.05); beta power increased at all six channels (*p* < 0.01); and gamma power increased at Cz, Pz, C3, C4, and F4 (*p* < 0.01), and at F3 (*p* < 0.05). In contrast, the theta power at F3 (*p* < 0.01) and F4 (*p* < 0.05) was significantly lower in the postdining condition.

Additionally, a significant Band × Stimulus Type interaction was observed at several channels, including Cz (*F*(3, 63) = 5.71, *p* < 0.01), Pz (*F*(3, 63) = 10.88, *p* < 0.01), C3 (*F*(3, 63) = 4.23, *p* < 0.01), and C4 (*F*(3, 63) = 3.99, *p* < 0.05). Post-hoc analyses showed that this interaction was primarily driven by the alpha band, where food images elicited significantly lower RP than neutral images in these four channels (*p* < 0.01). No significant differences were observed in the theta, beta, or gamma bands (*p* > 0.05), indicating that the neural response to food-related visual stimuli was selective to the alpha frequency range— likely reflecting alpha desynchronization, a well-established marker of increased cortical arousal and active attentional allocation to salient cues—and most prominent over central and parietal regions.

#### 2.2.4 Behavioral performance during the food probe task

**Figure 5:**
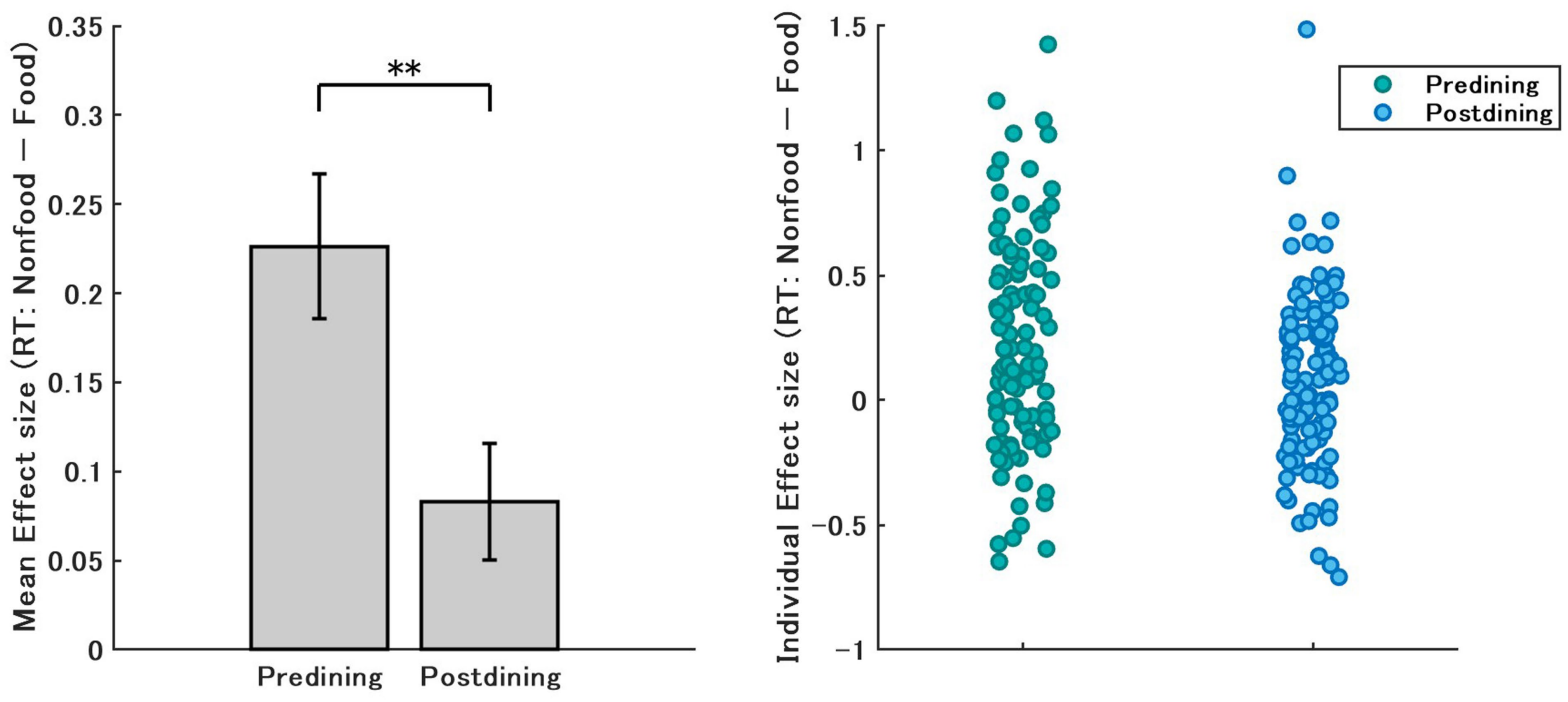
Mean and individual effect sizes of reaction time (RT) differences between nonfood and food stimuli in predining and postdining conditions. Left, mean effect size, with error bars showing the standard error of the mean. Right, individual effect sizes for each participant in both “predining” (green) and “postdining” (blue) conditions.

To evaluate the attentional bias toward food stimuli, we analyzed RTs for food versus nonfood images under predining and postdining conditions. Figure 5 shows the effect sizes for each participant calculated as the difference in RTs (nonfood– food), with positive values indicating faster responses to food. The results revealed significantly greater effect sizes in the predining condition than in the postdining condition (*p* < 0.01), indicating a stronger food-related attentional bias when participants were hungry. However, it should be noted that the absolute effect sizes in both conditions were relatively small. Under the postdining condition, effect sizes clustered closer to zero, suggesting that satiety dampens the attentional advantage for food stimuli.

**Figure 6:**
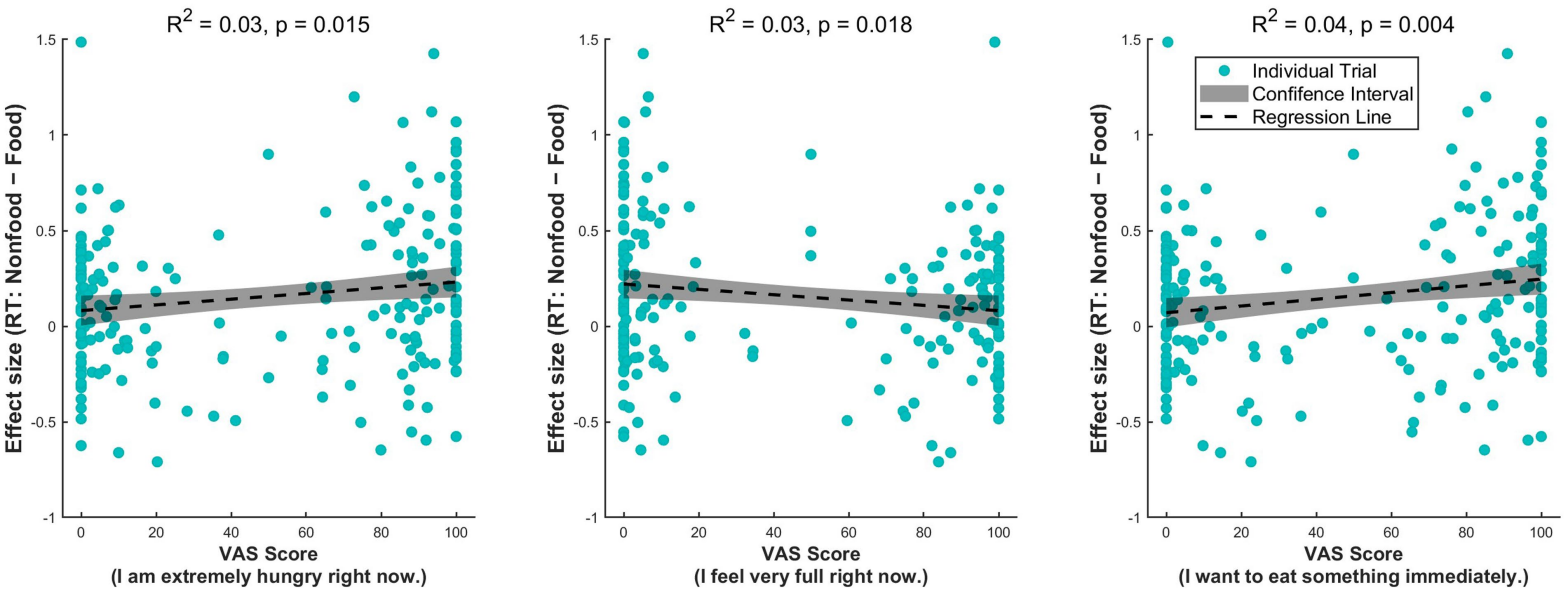
Scatter plots with regression lines showing the relationship between VAS scores and reaction time effect size (RT: nonfood–food). Each point represents one trial from a single participant. Shaded areas represent 95% confidence intervals.

To further explore the relationship between subjective hunger and the attentional response to food, we conducted regression analyses using VAS scores and RT effect sizes (Figure 6). Three VAS items were examined: (1) “I am extremely hungry right now,” (2) “I feel very full right now,” and (3) “I want to eat something immediately.”

Results revealed significant but weak correlations between subjective states and food-related RT bias. Specifically, the ratings of current hunger and desire to eat immediately were positively correlated with RT advantage for food stimuli (*R*^2^ = 0.03, *p* = 0.015; *R*^2^ = 0.04, *p* = 0.004, respectively), while fullness ratings showed a negative correlation (*R*^2^ = 0.03, *p* = 0.018). Importantly, these subjective feelings account for only 3–4% of the variance, indicating that the variance in RT is not well explained by subjective states alone.

## 3 Experiment 2

In Experiment 1, we observed that the postmeal satiated state is characterized by distinct neural signatures. During rest, there was a robust whole-brain increase in beta/gamma power (24–36 Hz), alongside a non-significant visually pronounced peak in the low-alpha band (~9 Hz). Importantly, during active food-cue processing, overall alpha power showed a significant elevation, which likely serves as a top-down inhibitory gate against food salience. Based on these combined resting-state trends and task-based significant findings, these specific frequency bands (beta/gamma and ~9 Hz low-alpha) were chosen as target components of “neuro-music.” In Experiment 2, we examined whether listening to this neural music under two conditions (predining and postsnack) can reproduce the subjective postmeal satiety observed in Experiment 1.

### 3.1 Materials and methods

#### 3.1.1 Musical stimulation

**Figure 7:**
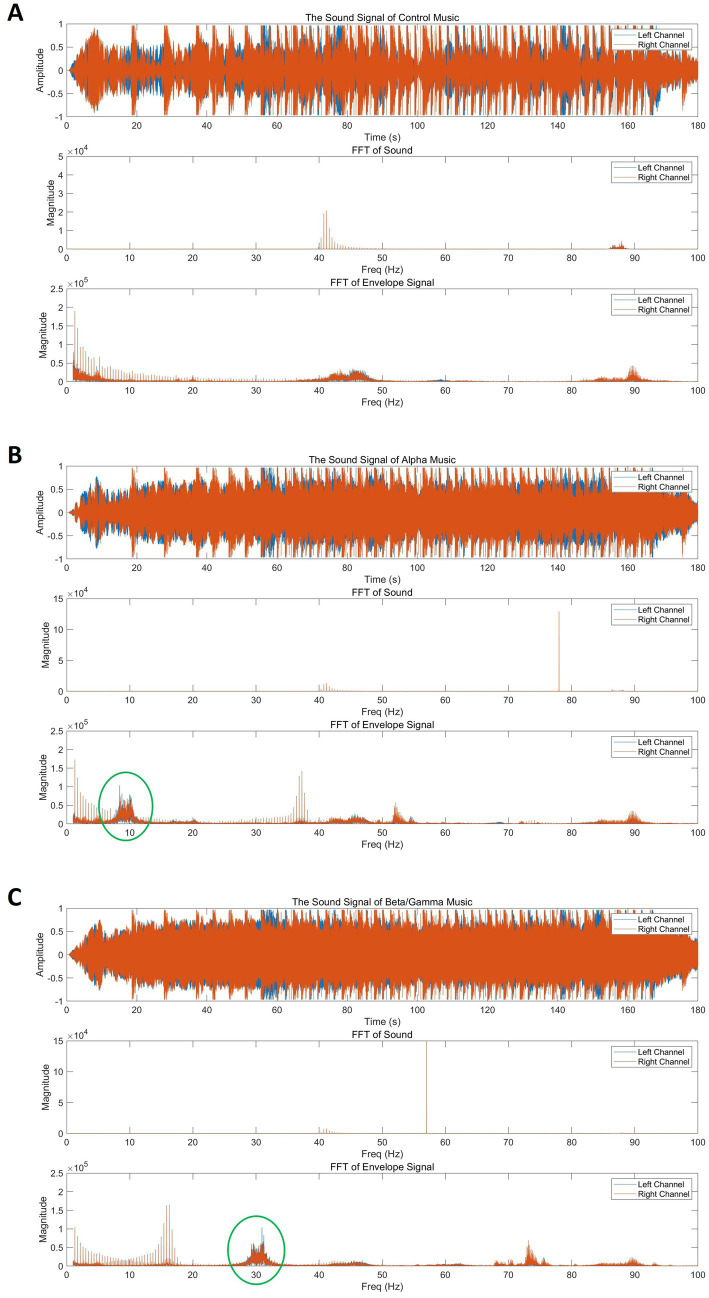
Audio signal analysis and frequency analysis of music tracks (**A**: Control; **B**: Alpha music; **C**: Beta/gamma music). Top, Waveform of a stereo music track showing the sound amplitude over 180 s for the left (blue) and right (orange) channels. Center, Fast Fourier Transform of the sound signal, revealing the musical frequency components in both channels within the 0–100 Hz range. Bottom, Frequency response of the envelope. Highlighted areas in the frequency domain (circled in green) indicate significant power peaks in the musical envelope.

Musical stimuli were composed using Ableton Live and included three versions: (1) a control track (control music, Figure 7A), (2) an alpha-band stimulation track (alpha music, Figure 7B), and (3) a beta/gamma-band stimulation track (beta/gamma music, Figure 7C). The control track was designed to emphasize the frequency band between 83–91 Hz, using the Drifting Ambient Pad synthesizer in Ableton Live as main sound source. Rhythmic elements were added with percussive sounds of acoustic characteristics outside the 83–91 Hz range. The alpha-band stimulation audio was designed by superimposing a 78 Hz sine wave onto the control audio. This was intended to shift the acoustic energy peak from 83–91 Hz into the alpha band (8–12 Hz), thereby producing monaural stimulation across the alpha frequency range (Chang et al., 2024). In the beta/gamma-band stimulation audio, a 57 Hz sine wave was superimposed onto the control audio. Because the primary frequency band of the control track’s synthesizer pad is between 83 and 91 Hz, adding the 57 Hz tone generated amplitude modulation (monaural beats) in the range of 26–34 Hz (i.e., the difference between the fundamental frequencies). This effectively created a stimulation envelope targeting the beta/gamma frequency range. The additional envelope peaks observed below 20 Hz and around 75 Hz represent the inherent rhythmic and acoustic components (e.g., percussive sounds) of the underlying control track, rather than explicitly targeted entrainment frequencies.

#### 3.1.2 Participants

A total of 600 healthy adults participated in the experiment. Participants ranged in age from 20 to 59 years (*M* = 40.3), with 298 males and 302 females. The average body mass index (BMI) across the sample was 21.27; most participants were within the normal weight range. The inclusion and exclusion criteria for this online study were consistent with Experiment 1, ensuring that all participants were aged 20–59 with normal hearing and had no self-reported history of eating disorders, severe obesity (BMI ≥ 30), metabolic diseases, psychiatric/neurological conditions, or current use of psychotropic medications. Recruitment and data collection were conducted exclusively online, and participants completed all tasks remotely while continuing their daily routines. All responses were submitted using personal devices such as smartphones, tablets, or personal computers. Informed consent was obtained electronically before participation and the study protocol approved by the relevant institutional ethics committee.

Since not all participants completed every session, we screened the dataset for completeness. For a given condition, data were considered valid if the participant had finished ≥2 sessions under that condition.

- Hungry condition: 306 participants (mean age = 40.69 ± 10.54 years), including 170 women; mean BMI = 20.96.
- Postsnack condition: 330 participants (mean age = 40.70 ± 10.41 years), including 180 women; mean BMI = 20.99.

#### 3.1.3 Procedure

**Figure 8:**
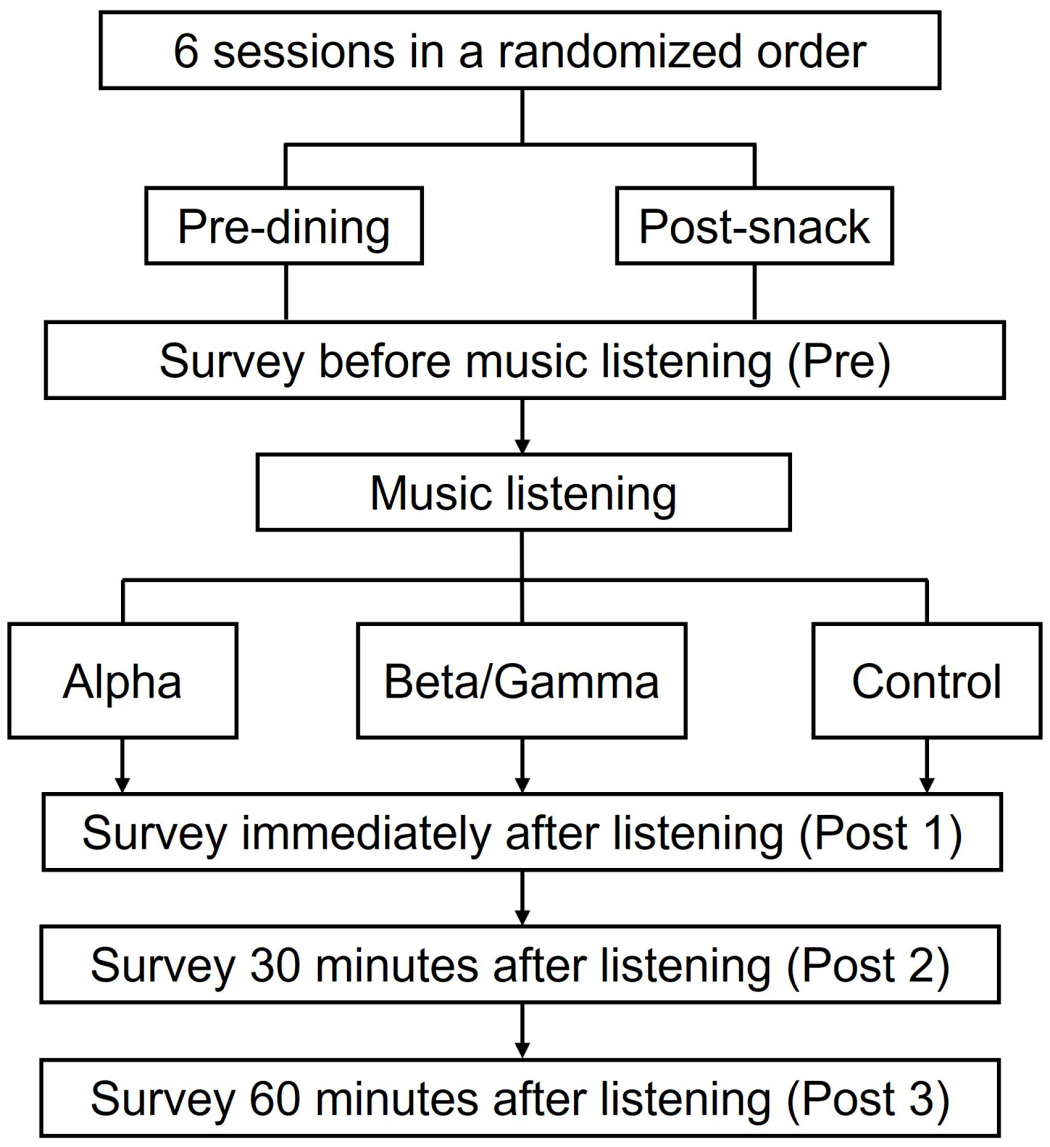
Overview of the experimental structure and time course for experiment 2, including the sequence of surveys and music exposure in hungry and postsnack conditions.

This experiment aimed to evaluate whether the neuro-music, developed based on the EEG frequency bands identified in Experiment 1, could influence subjective hunger and satiety under everyday settings. Participants completed six experimental sessions over six separate days, reflecting a 2 (physiological state: predining vs. Postsnack) × 3 (music type: alpha, beta/gamma, control) within-subject design (Figure 8). Each participant participated in all six conditions, but the sessions were not necessarily conducted on consecutive days. The order of conditions was randomized, and sessions could be flexibly spaced across different days.

During each session, participants completed hunger and fullness ratings at four time points: before music listening (Pre), immediately after (Post 1), 30 min (Post 2), and 60 min after (Post 3). These assessments were performed using self-report surveys based on a 10-point Likert scale version of the same VAS items used in Experiment 1, including statements such as “I am extremely hungry right now,” “I feel very full right now,” and “I want to eat something immediately.”

For patients, sessions began by completing the Presurvey before being instructed to listen to a short music stimulus (~2 to 3 min) through head or earphones while seated with their eyes closed. Then, they completed the three follow-up surveys at the designated time intervals.

Sessions were conducted under two physiological conditions: predining and postsnack. In the first, participants completed the session roughly 1 h before a meal, refraining from any caloric intake during the survey window. In the postsnack condition, they consumed a self-selected snack after the premusic rating and were asked to use the same snack and quantity across all three relevant sessions. Similarly, they were instructed to abstain from additional food or caloric drink intake during the assessment period.

#### 3.1.4 Statistical analysis

To evaluate the immediate effects of auditory stimulation on subjective hunger and satiety, we conducted a two-way repeated-measures ANOVA for each physiological condition (predining and postsnack) separately. The model included time (Pre vs. Post 1) as within-subject factor and music type (alpha, beta/gamma, control) as between-condition factor. The dependent variable was the VAS score reflecting subjective hunger. If a significant main effect or interaction involving music type was detected in the initial analysis, we assessed the potential sustained effects of auditory stimulation by conducting a second two-way repeated-measures ANOVA, separately for the predining and postsnack conditions. This model included time (Post 1, Post 2, Post 3) as a within-subject factor and music type as a between-condition factor, with VAS scores as the dependent variable.

A significance level of *p* < 0.05 was used for all statistical tests. Where appropriate, post-hoc pairwise comparisons were conducted using the Tukey–Kramer method to adjust for multiple comparisons.

### 3.2 Results

#### 3.2.1 Resource identification initiative

Immediate effects in subjective hunger were analyzed by separate 3 (Music: control, alpha, beta/gamma) × 2 (Time: Pre, Post 1) repeated-measures ANOVAs for each VAS item at predining and postsnack conditions.

**Figure 9:**
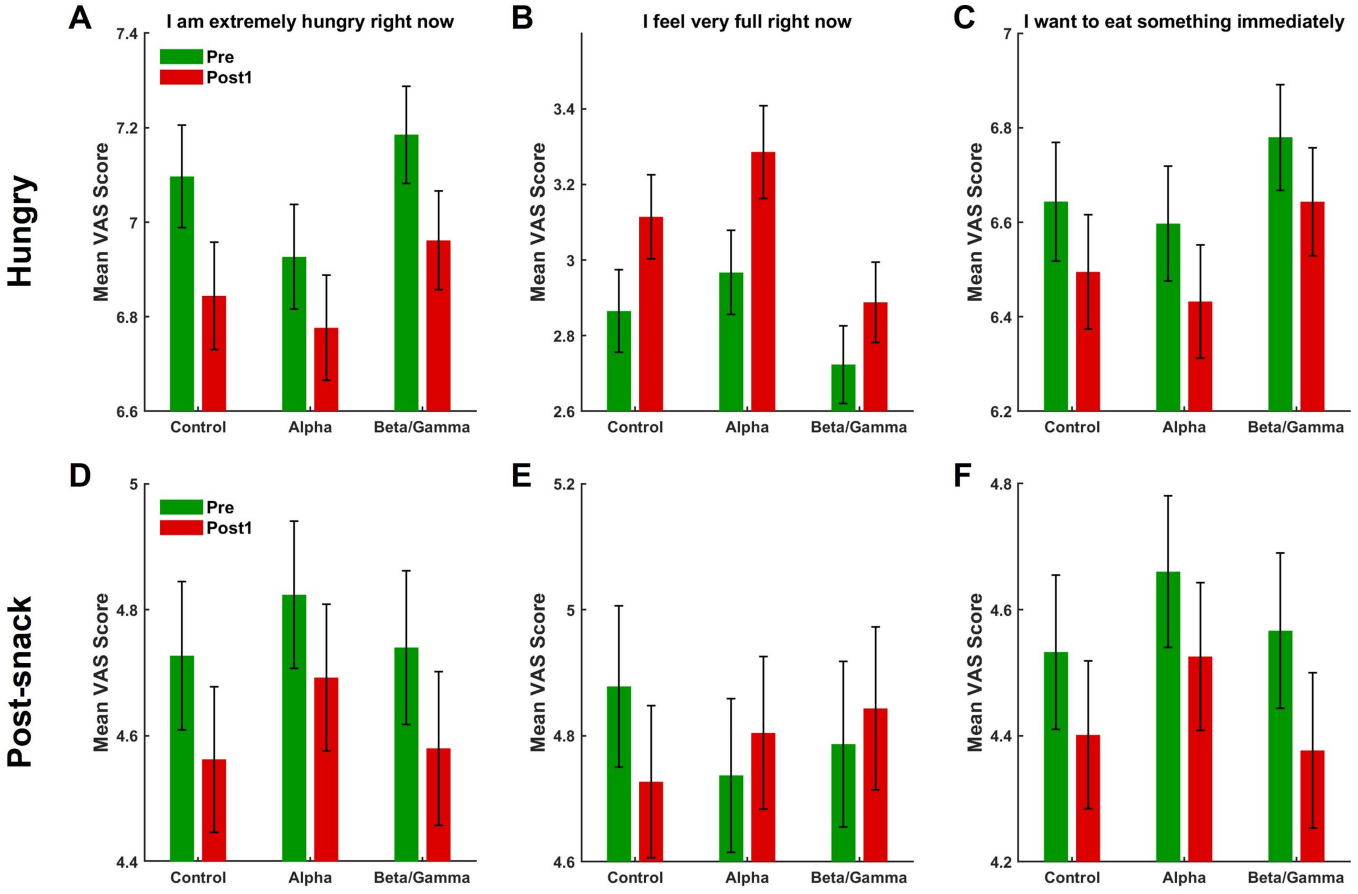
Mean VAS score before (Pre) and after (Post 1) music listening for three appetite-related items across music conditions, at hungry and postsnack states. **(A–C)** Results under the hungry condition showing changes in subjective ratings of **(A)** “I am extremely hungry right now,” **(B)** “I feel very full right now,” and **(C)** “I want to eat something immediately.” **(D–F)** Show corresponding measures under the postsnack condition for the same items. Green bars represent premusic score and red bars postmusic score. Music conditions include control, alpha, and beta/gamma music. Error bars indicate ±1 SEM.

In the predining state (Figures 9A–C), all three VAS items exhibited a significant main effect of time. Listening to music produced a statistically significant shift in subjective appetite ratings in the predining condition. Specifically, mean scores for “I am extremely hungry right now” (Figure 9A) and “I want to eat something immediately” (Figure 9C) both showed a significant decrease after music exposure (A: *F*(1, 845) = 35.78, *p* < 0.001; C: *F*(1, 845) = 13.04, *p* < 0.001), whereas the score for “I feel very full right now” (Figure 9B) increased significantly (*F*(1, 845) = 43.24, *p* < 0.001). However, there was no significant main effect of music type on any of the three items (A: *F*(2, 845) = 1.03, *p* = 0.36; B: *F*(2, 845) = 2.10, *p* = 0.12; C: *F*(2, 845) = 0.73, *p* = 0.48), suggesting that control, alpha, and beta/gamma music had comparable effects on hunger, appetite, and fullness. The absence of a significant interaction between time and music type (A: *F*(2, 845) = 0.75, *p* = 0.47; B: *F*(2, 845) = 1.43, *p* = 0.24; C: *F*(2, 845) = 0.04, *p* = 0.96) further indicates that the observed immediate changes occurred uniformly across music conditions.

In the postsnack state (Figures 9D–F), a main effect of time was observed for two of the three VAS items. Specifically, participants reported significantly lower scores after listening to music for “I am extremely hungry right now” (*F*(1, 913) = 15.25, *p* < 0.001) and “I want to eat something immediately” (*F*(1, 913) = 14.65, *p* < 0.001), but no significant change was observed for the item “I feel very full right now” (*F*(1, 913) = 0.03, *p* = 0.82). The slight visual decrease in fullness observed in the control group (Figure 9E) was not statistically meaningful, reflecting normal baseline fluctuation rather than a true increase in appetite. Nonetheless, no main effect of music type was found for any item (D: *F*(2, 913) = 0.27, *p* = 0.76; E: *F*(2, 913) = 0.03, *p* = 0.97; F: *F*(2, 913) = 0.35, *p* = 0.70), nor were there any significant time × music interactions (D: *F*(2, 845) = 0.07, *p* = 0.93; E: *F*(2, 845) = 1.84, *p* = 0.16; F: *F*(2, 845) = 0.23, *p* = 0.80).

#### 3.2.2 Sustained effects of music on subjective appetite ratings

To assess the persistence of music-induced effects on hunger and satiety, we conducted a series of 2 (time: Post1, Post2, Post3) × 3 (music type: Control, Alpha, Beta/Gamma) repeated-measures ANOVAs using normalized VAS ratings (Post1 values subtracted as baseline).

**Figure 10:**
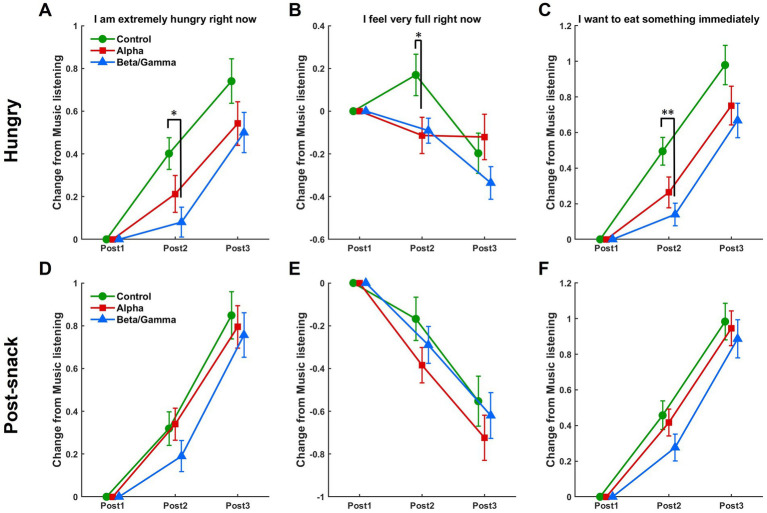
Sustained effects of music on subjective hunger and satiety ratings over time. Changes in VAS scores from baseline (Post1) for each music condition—control (green), alpha (red), and beta/gamma (blue)—at 30 min (Post2) and 60 min (Post3) after music listening. **(A–C)** Results for the hungry condition for **(A)** “I am extremely hungry right now,” **(B)** “I feel very full right now,” and **(C)** “I want to eat something immediately.” **(D–F)** Corresponding results for the postsnack condition. Error bars indicate ±1 SEM (^∗^*p* < 0.05; ^∗∗^*p* < 0.01).

In the predining state (Figures 10A–C), for the item “I am extremely hungry right now” (Figure 10A), an ANOVA revealed a marginally significant main effect of music type (*F*(2, 845) = 2.85, *p* = 0.059), and a marginal significance of the Time × Music interaction (*F*(4, 1,690) = 2.04, *p* = 0.086). To further explore this interaction, post-hoc pairwise comparisons were performed. At Post2 (30 min after listening), a significant difference was found between the Beta/Gamma and Control conditions, with Beta/Gamma showing lower values than Control (mean difference = −0.321, *p* = 0.012, 95% CI [−0.584, −0.058]), indicating that Beta/Gamma music produced a stronger, sustained reduction in hunger than the Control condition. No other significant comparisons were observed at this time point, nor were group differences at Post3 (60 min postlistening). For the item “I feel very full right now” (Figure 10B), there was no main effect of music type, *F*(2, 845) = 1.51, *p* = 0.222, but a significant Time × Music interaction emerged (*F*(4, 1,690) = 3.07, *p* = 0.016). Follow-up tests showed significantly higher fullness ratings at Post2 in the Control condition compared to the Alpha condition (mean difference = 0.283, *p* = 0.049, 95% CI [0.001, 0.566]). No other comparisons reached significance at Post2, and no differences were found at Post3. For the item “I want to eat something immediately” (Figure 10C), the main effect of music type was significant (*F*(2, 845) = 3.89, *p* = 0.02), as was the Time × Music interaction (*F*(4, 1,690) = 2.43, *p* = 0.046). At Post2, pairwise comparisons revealed a significant difference between Beta/Gamma and Control (mean difference = −0.355, *p* = 0.004, 95% CI [−0.616, −0.094]), indicating a stronger suppressive effect on urge-to-eat in the Beta/Gamma condition. No significant differences were observed at Post3.

In the Postsnack state, no significant main effects or interactions were observed across any of the three items (Figures 10D–F).

## 4 Discussion

This study explored the neural and behavioral correlates of hunger and satiety as well as the potential of frequency-specific auditory stimulation (neuro-music) to modulate subjective appetite states. Across two experiments, we found consistent evidence that hunger and satiety are reflected in distinct oscillatory EEG patterns and that beta/gamma-band auditory stimulation can modestly influence appetite-related perceptions.

### 4.1 Cortical oscillatory signatures of satiety

Our resting-state data indicated that postprandial satiety is characterized by a broadband amplification of cortical beta- and gamma-band synchrony together with modest low-alpha enhancement. These spectral shifts align with distinct neurophysiological frameworks: specifically, gamma-band activity has been linked to reward consummation (Van Der Meer and Redish, 2009), whereas cortical oscillatory dynamics are increasingly recognized as mechanisms for top-down interoceptive integration and the predictive coding of internal homeostatic states (Seth and Friston, 2016).

Beta activity (∼15–25 Hz) has been proposed to encode the maintenance of the current sensorimotor or cognitive set, signaling the status quo when a goal state has been achieved (Engel and Fries, 2010). In contrast, gamma oscillations (>30 Hz) are often associated with local circuit synchrony supporting feature binding and reward prediction (Buzsáki and Wang, 2012). The concurrent elevation of both bands after eating may therefore reflect a dual process: beta signaling a stable homeostatic set-point and gamma indexing dopaminergic reward completion. Previous PET studies showed overlapping ventral-striatal activation for palatable food and highly pleasurable music (Blood and Zatorre, 2001) as well as for fasting-induced bias toward high-calorie cues (Goldstone et al., 2009). Thus, cortico-striatal loops that broadcast reward receipt could drive the observed widespread beta/gamma synchrony, consolidating the internal representation of satiety.

Although smaller, the observed 9 Hz alpha increase is consistent with inhibitory gating (Jensen and Mazaheri, 2010): once a caloric need is met, alpha-mediated suppression may dampen further visual or interoceptive input related to food, reducing distractibility by residual hedonic cues. Recent MEG work supports that alpha power in parieto-occipital regions increases to suppress irrelevant visual information (Bonnefond and Jensen, 2012; Jensen and Mazaheri, 2010). Our whole-brain alpha increase likely reflects a similar gating mechanism after feeding. Furthermore, the relative increase in high-alpha power (10–13 Hz) during hunger likely reflects a state of heightened vigilance and sensory readiness (alertness) directed toward foraging and detecting environmental food cues, which is subsequently dampened upon feeding. Crucially, our task-based EEG data strongly support this model. We observed that alpha power was significantly lower in response to food stimuli compared to neutral stimuli, regardless of the physiological state. This alpha desynchronization reflects a universal, stimulus-driven cortical arousal to evolutionary salient cues. However, after eating, the overall baseline alpha power increases, serving as a top-down gate that successfully dampens the behavioral impact of this bottom-up salience.

Frontal midline theta has been linked to motivational drive, craving, and cognitive control over reward (Knyazev, 2012; Scheeringa et al., 2008). Its reduction after lunch suggests a down-shift in the motivational salience of food. Intriguingly, deep-brain recordings have shown theta bursts in the nucleus accumbens during anticipatory but not consummatory feeding phases (Van Der Meer and Redish, 2009), mirroring our surface EEG findings. This transition highly implicates the Default Mode Network (DMN), which is crucial for monitoring internal bodily sensations and interoceptive awareness. Given that frontal theta power correlates negatively with DMN activity (Scheeringa et al., 2008), the reduction in theta post-meal may facilitate a large-scale network shift: moving away from external stimulus detection (Salience Network) and toward DMN-mediated internal visceral processing and satiety evaluation.

Together, these results support a “reallocation” model where postmeal neural dynamics transition from a hunger-linked theta-dominant, externally oriented mode to a beta/gamma-dominant, internally consolidated mode, with alpha acting as a gate to inhibit further food ingestion. Such a model resonates with predictive-coding views of interoception (Seth and Friston, 2016), wherein satiation decreases visceral prediction error, allowing for higher-frequency synchrony and stabilizing the newly achieved metabolic state. It is important to contextualize these high-frequency findings with the classical role of the lateral hypothalamus (LH). The LH promotes feeding behavior and is known to drive cortical arousal and fast-wave EEG (beta/gamma) during food-seeking or foraging states (Stuber and Wise, 2016; Carus-Cadavieco et al., 2017). However, the widespread cortical beta/gamma synchrony observed here after eating—and exogenously entrained to reduce appetite—likely reflects a distinct neural process. Rather than LH-driven foraging arousal, postprandial high-frequency synchrony is thought to reflect cortico-striatal reward consummation and the top-down updating of interoceptive states. While LH-driven fast waves initiate the drive to eat, this post-consummatory cortical synchrony appears to signal the termination of that drive (‘goal achieved’). By identifying beta/gamma increase as a robust correlate of fullness, our findings validate the rationale for embedding these frequencies into neuro-music. If these oscillations were not merely epiphenomenal but causally involved in signaling “goal achieved,” then recreating them exogenously may help tip interoceptive inference toward satiety—even in absence of caloric intake. Future work combining simultaneous EEG- functional Magnetic Resonance Imaging (fMRI) for targeting subcortical structures with deep-brain-profiled transcranial Alternating Current Stimulation (tACS) could directly investigate this causal chain.

### 4.2 Behavioral correlates: hunger-dependent attention to food cues

Our behavioral data reveal a significant, albeit weak, hunger-state modulation of attentional priority. While this general direction replicates classic attentional-capture findings in food research (Mogg et al., 1998; Nijs et al., 2010) and echoes neuro-imaging evidence that hunger amplifies insula-striatal responses to highly caloric cues (Goldstone et al., 2009), the small effect sizes suggest that subjective hunger alone is not the sole driver of attentional bias. Prior studies have noted that attentional capture by food is highly susceptible to individual trait differences, such as trait impulsivity, dietary restraint, and baseline body mass index (Nijs et al., 2010). Thus, while the physiological state shifts baseline priorities, complex individual and environmental factors likely account for a large portion of the remaining variance.

Furthermore, we found significant but weak correlations between subjective states and food-related RT bias, with subjective feelings of hunger and fullness accounting for only 3–4% of the variance. Behaviorally, this implies that the attentional prioritization of food cues operates largely as an automated, implicit process—likely an evolutionary survival mechanism driven by metabolic deficit (Berridge, 2012)—rather than being strictly governed by conscious, subjective appraisals of hunger.

Interestingly, rather than implying a global reduction in vigilance, our behavioral results show that satiety specifically neutralizes the attentional bias toward food cues, bringing the reaction time difference between stimulus types close to zero. The postdining state effectively reduces the motivational salience of food, a behavioral shift that perfectly aligns with the fulfilled metabolic need. (While Section 4.1 details the resting and task-based cortical signatures of this shift, these behavioral data independently confirm the suppression of bottom-up food cue salience.)

### 4.3 Efficacy of frequency-specific neuro-music

Building on evidence that rhythmic stimulation can entrain cortical oscillations (Baltus and Herrmann, 2015; Nozaradan, 2014; Notbohm et al., 2016), Experiment 2 tested neuro-music embedding either the alpha or beta/gamma components derived from Experiment 1. Immediately after music exposure, all conditions resulted in reduced hunger ratings; however, no significant group differences emerged at this stage, pointing to a general placebo or expectancy effect. This initial, undifferentiated decrease likely reflects nonspecific psychological mechanisms, such as relaxation-induced changes in arousal or attentional diversion (Blood and Zatorre, 2001; Bernardi et al., 2006), rather than frequency-specific neural entrainment. Indeed, previous research suggests that listening to music—irrespective of its spectral properties—can transiently influence emotional and physiological states, likely through generalized attentional diversion or calming effects (Juslin and Västfjäll, 2008; Koelsch, 2014). This generalized calming effect likely explains the unexpected finding in Figure 10B, where the ambient control track transiently increased subjective satiation. Such relaxation may promote a mild parasympathetic dominance (‘rest and digest’ state) and provide cognitive distraction from interoceptive hunger cues, thereby elevating baseline feelings of fullness even in the absence of targeted neural entrainment.

However, at the 30-min follow-up assessment, beta/gamma stimulation produced a modest yet statistically significant additional reduction in hunger and urge-to-eat ratings relative to the control. The delayed effect observed is unlikely to be attributable solely to an ongoing placebo or expectancy effect, as the initial broad reduction noted in the control group had subsided by this later assessment. Rather, the sustained and selective impact of beta/gamma neuro-music suggests that specific frequency entrainment indeed interacted with underlying neurophysiological processes to modulate interoceptive hunger signals. Such frequency-specific effects align well with the idea that high-frequency oscillatory synchrony, especially within beta and gamma bands, may play an active role in facilitating top-down updating of interoceptive predictive models and suppressing homeostatic hunger signals at subcortical levels (Seth and Friston, 2016).

The lack of sustained differentiation between alpha stimulation and the control further reinforces the idea that the alpha-band changes observed postprandially (Experiment 1) are consequences, and not causal factors, of satiety. While alpha-band oscillations are widely recognized for their role in attentional gating and inhibitory processing (Jensen and Mazaheri, 2010), the current results imply that experimentally enhancing alpha power alone may not suffice to induce satiety-like effects in the absence of actual caloric intake. Rather, the low-alpha enhancements observed after meals might basically reflect downstream inhibitory gating mechanisms activated after satisfaction of a physiological need, thus decreasing the motivational salience of external food cues (Klimesch, 2012).

Moreover, the absence of significant neuro-music effects in the postsnack condition strongly emphasizes state-dependency. With low physiological hunger and interoceptive prediction errors, additional entrainment of cortical oscillations may offer minimal incremental benefits, consistent with theoretical frameworks highlighting state-dependent modulation of reward and motivational circuits (Berridge, 2012). Practically, this indicates that beta/gamma neuro-music might be most effective in contexts of heightened metabolic need or craving, such as during extended fasting, dieting, or acute hunger episodes.

### 4.4 Limitations and future work

While the current study provides promising initial insights into the potential of neuro-music for appetite regulation, several important limitations warrant consideration, highlighting avenues for future research. First, the relatively short duration of neuro-music exposure (~3 min) and the transient nature of its effects raise questions regarding the sustainability and practical applicability of these interventions in real-world settings. Future studies should examine the effects of longer exposure times or repeated administration across multiple sessions to assess whether more enduring entrainment of cortical oscillations can result in sustained reductions of subjective hunger and food-seeking behaviors. Second, the lack of adequately validated neural data for Experiment 2 is another limitation. Although the behavioral outcomes suggest frequency-specific cortical entrainment, without sufficiently reliable concurrent EEG or neuroimaging data, the underlying neural mechanisms remain inferred rather than empirically demonstrated. Incorporating simultaneous EEG or neuroimaging to future studies would clarify how cortical entrainment directly relates to the observed behavioral outcomes. In addition, the strong placebo-like responses observed immediately after music exposure emphasize potential expectancy or psychological influences. Concurrent EEG measurements or other physiological indices (e.g., hormonal markers such as ghrelin, leptin, cortisol, and heart rate variability) during neuro-music listening could help differentiate genuine neurophysiological entrainment effects from psychological factors. Third, our cohort consisted primarily of healthy individuals, which limits the immediate transferability of these findings to clinical populations. As highlighted by Nijs et al. (2010), overweight and obese individuals often exhibit significantly different neural and behavioral responses to food cues, including heightened attentional biases, compared to normal-weight controls. While our findings offer a proof-of-concept, future research must specifically target these demographics to determine whether this non-invasive neuro-music can effectively modulate hyperactive food-cue reactivity in populations that would benefit most from such interventions. Finally, due to the constraints of our sample size and the relative homogeneity of our participants, we did not employ an Analysis of Covariance (ANCOVA) to control for factors such as age, sex, and specific anthropometric features (e.g., exact BMI). Given that these individual traits likely influence baseline interoception and reward processing, the absence of covariate control restricts our ability to reveal deeper individual-level correlations. Larger-scale future studies should systematically incorporate ANCOVA models to disentangle how these demographic and physiological features interact with the efficacy of acoustic cortical entrainment. Addressing these issues is essential for translating neuro-music interventions into practical tools for appetite control. Given the transient, 30-min window of efficacy observed here, real-world applications could involve strategic timing: for example, listening to neuro-music immediately prior to meals to acutely reduce portion sizes, or using it as a brief ‘craving-breaker’ to ride out sudden urges to binge or snack.

## 5 Conclusion

The present findings show that satiety is characterized by a cortex-wide increase in beta/gamma oscillations and a concomitant behavioral reduction in food-directed attention. Auditory stimulation designed to entrain these high-frequency rhythms modestly suppressed hunger under fasting conditions, suggesting a novel, noninvasive avenue for appetite regulation. By coupling neurophysiological insights with targeted acoustic design, “neuro-music” could contribute to integrative interventions for overeating and obesity.

## Data Availability

The original contributions presented in the study are included in the article/supplementary material, further inquiries can be directed to the corresponding author.
